# 93. Recurrent infections in a patient treated with biologic therapy for seropositive rheumatoid arthritis

**DOI:** 10.1093/rap/rky032.001

**Published:** 2018-09-20

**Authors:** Ameen Jubber, May Zun Swe, Rachel Jeffery

**Affiliations:** Rheumatology, Northampton General Hospital, Northampton, UNITED KINGDOM

**Introduction:** We present here a 38 year old female with a ten-year history of seropositive rheumatoid arthritis. Her treatment has included triple DMARD therapy, two variants of anti-TNF therapy and rituximab. During this time, she has suffered from two episodes of right hip septic arthritis and recurrent staphylococcus aureus chest infections, initially manifesting as cavitating lung nodules. During this time she was also managed through a pregnancy and developed complications from corticosteroids. This case highlights the challenging nature of trying to achieve adequate disease control to prevent deformities and disability, and treating recurrent complex and serious infections.

**Case description:** This 38 year old female patient was diagnosed with seropositive inflammatory arthritis at the age of 28. She was treated initially with triple DMARD therapy (methotrexate, sulfasalazine and hydroxychloroquine). She developed septic arthritis of the right hip two years later, requiring an open washout, although joint fluid cultures were negative. Three months post-operatively, she was back on triple DMARD therapy, but her inflammatory arthritis was flaring (DAS28 6.54). Anti-TNF therapy (adalimumab) was therefore commenced, with a plan to closely monitor the right hip radiologically for any signs of infection. This therapy was very effective in controlling the inflammatory arthritis. Three years later, the patient developed dyspnoea and a cough productive of green sputum with streaks of blood. A CT scan of the chest demonstrated multiple nodules in both lungs, some of which were cavitating. Sputum cultures grew *staphylococcus aureus* sensitive to flucloxacillin and clarithromycin. A bronchoscopy was performed, and washings were negative for acid fast bacilli and pneumocystic jiroveci (PCP). Cytology was negative for malignant cells. The methotrexate and anti-TNF therapy were held, but sulfasalazine continued (partly also because the patient expressed the wish to become pregnant soon). Although the respiratory symptoms improved with antibiotics, symptoms would flare soon after the antibiotic course ended. The antibiotics were continued long term on the advice of the microbiologist (and would continue until December 2016). The patient then became pregnant (complicated by gestational diabetes), and three months into the pregnancy she again developed a presumed septic arthritis of the right hip. An open washout was again performed, but joint fluid cultures were negative. Shortly after childbirth, her inflammatory arthritis was severely flaring and she was noted to be showing signs of ulnar deviation of the MCP joints. The patient was very keen to restart treatment as the arthritis was severely affecting her functional abilities. Anti-TNF therapy was therefore restarted, along with the prophylactic antibiotics to cover staphylococcus aureus. Etanercept was favoured this time over adalimumab owing to its shorter half-life. This was stopped after one year however due to reduced efficacy. After multiple courses of steroids to control the inflammatory arthritis, reduced mobility because of her joint problems and pregnancy, this patient suffered from weight gain that qualified her for bariatric surgery. Furthermore, as part of investigations for anaemia, she underwent an upper GI endoscopy that showed moderate to severe gastritis that precluded the use of further NSAIDs. In order to control her inflammatory arthritis, she was started on rituximab infusions in July 2016, alongside methotrexate and sulfasalazine. She had three courses of rituximab; each course providing around five months of symptom relief. Four months after the third course, however, she developed a cough productive of green sputum and streaks of blood. A repeat CT chest showed new bilateral bronchiectasis, as well as the old residual lung nodules, with no cavitation. Immunoglobulins were normal and an Immunology specialist opinion was sought as to whether there is any pre-existing immunodeficiency to account for these recurrent infections. Immune function studies were all normal with no evidence of additional risk factors other than her immunosuppressive therapy. She was seen in a combined chest-rheumatology clinic. Sputum clearance exercises were given, treatment for a recurrent *staphylococcus aureus* chest infection and then long-term antibiotics. She is hoping to try for another pregnancy and is starting on certolizumab.

**Discussion:** This case demonstrates the balancing act between treating infection and suppressing inflammation. Disease control in this patient is crucial, given the signs of deformity developing at a young age. However, the two episodes of septic arthritis and chronic recurrent *staphylococcus aureus* chest infections make this challenging. Notwithstanding the immunosuppressive aspects of treatment, this lady was affected adversely by corticosteroids and NSAID use as well. A multidisciplinary approach involving rheumatology, orthopaedics, respiratory, immunology and microbiology was used in this patient’s care. Following the first episode of septic arthritis, it was recognised that immunomodulatory therapy could mask the signs of sepsis, so serial imaging of the hip was used to ensure that a joint effusion is promptly identified. It was also important to ensure that an adequate period of time elapses after the infection resolves before starting the anti-TNF therapy. After the first septic arthritis, treatment was started after 12 months. After the CT chest, a wide range of differentials were considered for the cavitating lung lesions, including malignancy, vasculitis, rheumatoid nodules and infection, including TB reactivation following TNF inhibitor use (though the patient had a negative quantiferon test pre-treatment). Since sputum cultures were positive, the symptoms had improved and there was radiographic regression with antibiotics, staphylococcus aureus chronic recurrent infection was deemed the cause. One possible option is to use long term prophylactic antibiotics during immunosuppression, although this carries with it the risk of C. Difficile infection and risk of development of resistant organisms. Immunology input is important in cases such as this where infections are recurrent. Immunology investigations were normal, including immunoglobulins, B-cell function and neutrophil respiratory burst test. It was thought that she had secondary staphylococcal infection relating to rheumatoid associated cavitating lung disease.

**Key Learning Points**: Manage these patients with a multidisciplinary team approach. Patients on biologics may not manifest typical signs of infection or sepsis, a high index of suspicion is needed to identify infection early. Consider prophylactic antibiotics in patients with chronic infections where cultures and sensitivities are known.

Where infections are recurrent, consider other causes of immune dysfunction. A new biologic option is available during pregnancy.

**Disclosure: A. Jubber:** None. **M. Zun Swe:** None. **R. Jeffery:** None.

